# The agricultural carbon reduction effect of digital rural construction under the dual carbon target

**DOI:** 10.1371/journal.pone.0299233

**Published:** 2024-04-10

**Authors:** Haihong Guo

**Affiliations:** School of Economics and Management Department, Qingdao Agricultural University, Qingdao, China; Abdelmalek Essaadi University: Universite Abdelmalek Essaadi, MOROCCO

## Abstract

The exploration of the agricultural carbon emission reduction effect of digital rural construction offers a promising path towards achieving dual carbon goals. This study establishes an evaluation system for digital rural construction and analyzes its impact on agricultural carbon emissions using various creative techniques including panel fixed effects, mediation effects, threshold effects, and spatial Durbin models based on provincial panel data from 2011 to 2021.It is found that: (1) The impact of digital rural construction on agricultural carbon emissions exhibits a "inverted U-shaped" pattern, with a nonlinear effect on emissions through promoting agricultural green total factor productivity and adjusting agricultural structure.(2) Digital rural construction has both promoting and inhibiting effects on agricultural carbon emissions, both locally and in adjacent areas. It also demonstrates a threshold effect, with rural human capital as the sole threshold. Once the threshold value 8.830 is surpassed, the agricultural carbon emission reduction effect becomes prominent.(3)Digital rural construction has a dual effect on local agricultural carbon emissions in terms of both promoting and then restraining the emissions, which has a spatial spill-over effect in the neighboring areas. This study contributes to our understanding of carbon reduction pathways by highlighting the comprehensive utilization of digital rural construction and expanding research on the dynamic context of its impact on carbon emissions.

## 1 Introduction

To alleviate the immense pressure of global climate change, China has proposed the dual carbon goals of "peak carbon dioxide emissions" and "carbon neutrality". Agriculture, being a significant source of carbon emissions, is a key focus area for achieving these goals. It accounts for approximately 25% of the total carbon emissions in China, making it the second-largest source of carbon emissions after the industry [1].Within China, the agricultural and rural sectors have contributed to around 15% of the country’s total greenhouse gas emissions, including carbon dioxide [2]. To address this, various policy plans, such as the Action Plan for Carbon Peaking before 2030, have been issued and implemented since 2021, promoting carbon peaking actions in the agricultural sector. These policies have resulted in significant achievements in reducing agricultural carbon emissions, including both the total amount and intensity of emissions [3].However, certain factors hinder the green, low-carbon, and high-quality development of agriculture. These factors include low agricultural production efficiency, excessive use of agrochemicals, land fragmentation, and inefficient resource allocation, which directly or indirectly contribute to increased agricultural carbon emissions and higher carbon emission intensity [4]. Therefore, it is crucial to explore and address the key influencing factors of agricultural carbon emissions.

Existing literature has examined several factors that influence agricultural carbon emissions, including farm size, urbanization, carbon reduction policies, agricultural mechanization, and technological progress. Studies have found that larger farm sizes tend to increase agrochemical input intensity and induce changes in production techniques [5]. As rural populations migrate to urban areas and agricultural land decreases, the intensity of agricultural carbon emissions may decrease. However, urbanization also brings about changes in agricultural production patterns and promotes agricultural mechanization, which can lead to increased carbon emissions. Urbanization has a significant and lasting impact on the total amount of agricultural carbon emissions [6]. The expansion of mechanization in agriculture can result in increased soil cultivation demands and greater use of agricultural chemicals, thereby affecting agricultural carbon emissions [3]. Strategies for reducing carbon emissions in agriculture can be guided by low-carbon special plans, government support, and interventions targeting agricultural households [7].Technological progress plays a crucial role in improving carbon emission reduction efficiency, whereas efficiency improvement alone may not be sufficient [8]. Advanced green technologies have positively influenced the enhancement of agricultural carbon emission reduction efficiency in various regions. Long-term strategies to promote low-carbon agriculture involve the implementation of policies combining incentives and regulations. However, traditional rural construction does not necessarily lead to green technological progress or drive agricultural industrial restructuring. Further research and exploration are still needed to discover more effective mechanisms for reducing agricultural carbon emissions.

At the same time, digital rural construction has been fully rolled out in China, with notable emphasis on its promotion and implementation through the "Outline of digital rural construction Strategy" and the "Number One Central Document" for 2019–2023. These initiatives have contributed to accelerating the progress of digital rural construction. As of 2021, the level of digitalization in agricultural production has reached 22.5% [9]. It is worth noting that digital rural construction has played a crucial role in driving industrial green transformation and encouraging low-carbon consumption by residents. Moreover, it has increasingly become a new engine for promoting greening, low-carbonization, and sustainable development in agriculture [10].The essence of digital rural construction is to integrate the digital economic paradigm with the agricultural and rural economy, aiming to enhance resource allocation efficiency, reduce operational costs, and introduce new agricultural industry models [11].It also facilitates agricultural transformation, comprehensive rural development [12], and improves the lives of farmers [13]. Despite significant progress in digital infrastructure, agriculture digitalization, and digital governance in rural areas [14], challenges such as the digital divide and regional imbalance persist [15].Resolving these issues and promoting high-quality digital rural area construction remain important concerns. While existing research explores the current situation, challenges, and strategies for digital rural construction, there is a lack of consensus on evaluating its effectiveness and defining a standardized indicator system.

Existing literature has shown that digital rural construction can have positive impacts on the agricultural sector. It can lead to an increase in total factor productivity in agriculture [16], contribute to the reduction of chemical inputs such as fertilizers and pesticides, improving sustainability in agricultural practices [17]. Additionally, it can facilitates the emergence of new industries like digital finance and digital agriculture, which can further promote carbon emission reduction in agriculture [18–20].However, there is currently no consensus on the specific impact of digital rural construction on carbon emission reduction in agriculture, particularly in the context of dual carbon goals. The theoretical logic and empirical evidence surrounding the driving effects of digital rural construction on agricultural carbon emissions through green technological progress and industrial restructuring are still unclear. Additionally, the varying levels of digital rural construction across different regions, with some areas lacking complete hardware facilities and software conditions, pose challenges and room for improvement in empowering digital rural construction for agricultural production. This study aims to address this issue. Table 1 summarizes the research method, data type, and sample period in the above literature.

**Table 1:** Current literature review on the impact of digital rural construction on carbon emissions.

Authors	Sample Period	Data Type	Methos	Results
Jin S R and Ren Z J.(2022)	2011–2020	Panel data	EBM	rural digitalization↑→ agricultural green total factor productivity
Du J J et al.(2023)	2019	1740 sample	mediation effect model	digital village construction↑→ agricultural green total factor productivity
Yang X and Zhao S G(2022)	2006–2019	Panel data	the stirpat equation	digital economy↓→carbon emission
Chen Z W and Tang C(2023)	2010–2020	Panel data	a regression model	digital economy↓→ agricultural carbon emissions;
Liu Z et al. (2024)	2013–2020	Panel data	a regression model	rural digital economy ↓→ agricultural carbon emissions
Fu J H and Xue J X.(2024)	2014–2021	Panel data	the fixed effect model	the construction of digital villages ↑→ the green development of agriculture

Given the limitations observed in previous literature, this paper seeks to comprehensively analyze the effects and mechanisms of digital rural construction on agricultural carbon emissions from multiple dimensions using provincial panel data from 2011 to 2021, by employing various advanced analytical techniques such as panel fixed effects, mediation effects, threshold effects, and spatial Durbin models.This paper aims to provide a more nuanced understanding of the relationship between digital rural construction and agricultural carbon emissions, shedding light on the underlying mechanisms and potential threshold effects.

In comparison to previous studies, this research contributes in three aspects: Firstly, it fully considers the interconnections between digital infrastructure, agricultural digitization, and rural digitization, constructing an integrated evaluation system for the level of digital rural construction that incorporates "digital infrastructure-agriculture-rural" elements. This provides a measurement standard for assessing the effectiveness of digital rural construction. Secondly, based on the essential attributes of digital rural construction and the fundamental characteristics of agricultural carbon emissions, this study clarifies the marginal effects and mechanisms of digital rural construction on agricultural carbon emission reductions from various dimensions such as technological effects and structural effects. Furthermore, it extends the analysis to include the threshold effects of rural human capital, providing theoretical foundations for the transformation and upgrading of agricultural green low-carbon development. Lastly, this research fully considers spatial correlation and examining it from both direct and indirect spill-over perspectives offer valuable insights for the formulation of policies promoting regional agricultural green development. These contributions enhance our understanding of digital rural construction and offer valuable insights for policy formulation in the realm of regional agricultural green development.

## 2 Literature review and research hypotheses

### 2.1 The direct impact

Digital rural construction aims to improve agricultural productivity, living conditions in rural areas, digital literacy among farmers, and and foster internal dynamics for agricultural and rural development by integrating modern information technology with agricultural practices and rural lifestyles [21]. From a theoretical perspective, both digital economic development and agricultural carbon emissions, on which digital rural construction depends, have a non-linear characteristic. According to Metcalfe’s law, there is an exponential growth relationship between network effects and the number of users [22]. When digital rural construction reaches a certain critical value, the positive feedback mechanism triggered by rural network users would promote a sudden growth in rural network value. According to the "China Internet Development Status Statistical Report," as of December 2021, the number of rural netizens has reached 284 million, and the Internet penetration rate in rural areas has reached 57.6%,which is indicating enormous potential for continued growth. Based on the Environmental Kuznets Curve (EKC) theory, there is a non-linear relationship between agricultural environmental pollution and agricultural economic development [23]. It is likely that the combination of digital rural construction and agricultural carbon emissions would also exhibit a non-linear "inverted U-shaped" relationship.

The impact of digital rural construction on agricultural carbon emissions is a topic of debate. Proponents argue that digital rural construction can contribute to reducing agricultural carbon emissions. It can create a digital ecosystem that promotes ecological environment improvements, reduces energy consumption in agricultural production, minimizes damage to rural production and living environments, and subsequently lowers agricultural carbon emissions [24].On the other hand, critics argue that digital rural construction may have a suppressing effect on agricultural carbon emissions. They highlight that the implementation of digital infrastructure to support rural development requires significant investment, which in turn consumes resources and increases energy consumption levels [25].This could potentially result in energy displacement effects, placing a burden on the ecological environment and leading to increased agricultural carbon emissions. Overall, the relationship between digital rural construction and agricultural carbon emissions is a complex issue that requires further research and analysis to gain a comprehensive understanding of its impact.

Thus, digital rural construction has both suppressing and promoting effects on agricultural carbon emissions. In the short term, the improvement of communication technology in digital rural construction will increase diesel and electric energy consumption, leading to total agricultural carbon emissions increases. However, in the long run, digital rural construction promotes factor innovation and the substitution of high-energy-consuming energy, improving factor allocation efficiency and green production efficiency, thus reducing agricultural carbon emissions. Given this, Hypothesis 1 proposes that the impact of digital rural construction on agricultural carbon emissions follows an "inverted U-shaped" trend.

### 2.2 Mediating effects

#### 2.2.1 Technological progress

According to the induced technological progress theory, the penetrability and replicability of the digital economy can contribute to agricultural technology research and green technology innovation, thereby driving green technology progress [26].The theory proposes four mechanisms through which digital rural construction impacts agricultural carbon emissions:(1)Estructuring effect: The integration of data as a new production factor with traditional agricultural factors leads to improved resource utilization efficiency, reduced agricultural carbon emissions, and increased marginal return rate of labor, land, and capital [27]. (2)Pulling effect: Optimizing resource allocation and reducing waste and redundancy in agricultural production processes helps promote technology upgrades, reducing dependence on traditional factors and subsequently reducing agricultural carbon emissions [8]. (3)Integration effect: By integrating digital technology with agricultural big data, the marginal substitution rate between factors is changed, enabling better precision in agricultural technology guidance and promotion. This promotes voluntary environmental regulation, increases acceptance of green technologies, and decreases agricultural carbon emissions [28]. (4) Permeation effect: The continuous improvement of digital infrastructure allows advanced technologies like 5G, artificial intelligence, and cloud computing to penetrate the agricultural sector. This enhances data sharing efficiency, improves the precision of agricultural technology R&D, lowers marginal costs of new agricultural technologies, and improves factor utilization efficiency, ultimately reducing agricultural carbon emissions [1].Given this, Hypothesis 2 suggests that digital rural construction has a positive technological effect, promoting green technology progress and reducing agricultural carbon emissions. However, it’s important to note that the impact of green technology transformation and efficiency improvement on agricultural carbon emissions may differ.

#### 2.2.2 Agricultural structure adjustment

According to the industrial structure theory, agricultural structure transformation is essentially the change in industry and factor share brought about by the flow and reconfiguration of factors between industries, and the degree of share change depends on the difference in the skill-biased technological change growth rate between industries [29]. Digital rural construction can promote the transformation of agricultural structure, driving it towards rationalization and upgrading. Firstly, digital rural construction can accelerate the circulation of agricultural information, breaking down information barriers. Agricultural operators can use digital technology to quickly acquire information on production experience, production technology, market demand, government policies and other aspects, reasoably allocate resources, facilitate timely adjustment of production structure, quickly connect with the agricultural market, and reduce agricultural carbon emissions [30].At the same time, digital rural construction based on digital technology can break through information barriers between urban and rural areas and regional markets, reduce information search costs, promote rationalization of industrial structure, reduce loss rate, and reduce carbon emissions [31]. Secondly, digital rural construction is conducive to optimizing agricultural production processes. The key task of digital rural construction is to achieve digitized operation of the full process of agricultural production, operation, and sales. By driving process transformation to promote deep-seated changes in the "Internet+" model in agricultural product processing, packaging, circulation and other links [16], it can promote the upgrading of industrial structure, improve the efficiency of green technology, and reduce carbon emissions. Thirdly, digital rural construction can promote the advancement of agricultural industrial structure towards a higher level through the strong penetrability of digital technology, breaking down the boundaries of agricultural industry [32]. At the same time, with the expansion of the agricultural operators’ social network and the improvement of their digital literacy, they have higher demands for quality, which promotes the adjustment of agricultural input factor structure and reduces the input of high-energy and high-polluting factors, thereby reducing agricultural carbon emissions. Based on the above, hypothesis 3 is proposed: Digital rural construction can influence agricultural carbon emissions by promoting the upgrade of agricultural structure.

### 2.3 The spatial spillover effect

According to spatial dependence theory, digital rural construction and agricultural production processes are considered open systems. Digital rural construction facilitates the flow of factors and the diffusion of green agricultural production technology, thereby promoting spatial interaction and generating spillover effects. These spillover effects can manifest as both tangible and intangible knowledge. Tangible knowledge refers to the knowledge embodied in physical entities such as green high-quality breeds, digital technology equipment, and green organic materials. On the other hand, intangible knowledge refers to knowledge embedded in digital thinking, green concepts, digital production technology, and other non-physical carriers that permeate the digital rural construction process [2].The spatial spillover effects of digital rural construction on agricultural carbon emissions can be attributed to several factors. Firstly, digital rural construction fosters agglomeration effects. It relies on digital technology support and a mature market for digital technology cultivation. Regions with locational, industrial, and resource advantages are more conducive to the concentration of digital factors, leading to agglomeration effects. This, in turn, facilitates the absorption of green, low-carbon tangible and intangible knowledge, promotes spatial interaction, and generates demonstration effects. Secondly, digital rural construction leads to diffusion effects. Drawing on the "center-periphery" theory, the green and low-carbon demonstration effects facilitated by digital rural construction spread between adjacent regions, different agricultural fields, and agricultural operators. This, in turn, promotes competition, learning, and imitation, creating a "snowball effect" and driving regional agricultural carbon reduction. Thirdly, digital rural platforms enable knowledge exchange and sharing between different regions, agricultural industries, and management entities. This facilitates the resolution of challenges and difficulties in low-carbon development, leading to the creation of regional "group effects" and further expanding spillover effects. Therefore, hypothesis 4 is proposed: digital rural construction has spatial spillover effects on agricultural carbon emission reduction.

### 2.4 Threshold effect of rural human capital

The effective implementation of carbon emission reduction in digital rural construction is closely related to the digital literacy of agricultural operators [9]. When the level of rural human capital is relatively high, it provides intellectual support for agricultural management. This enables a better understanding of production laws, leading to precise input of pesticides and fertilizers, ultimately promoting carbon emission reduction in agriculture. Furthermore, higher levels of digital literacy make it easier to grasp market signals during agricultural product operations, reducing unnecessary input losses and carbon emissions. Digital economic forms such as e-commerce and live streaming eliminate information asymmetry in the sales market, reducing agricultural product losses, expanding green agricultural product sales channels, and further reducing carbon emissions. However, when the level of rural human capital is relatively low, it becomes challenging to provide intellectual support to agricultural management entities. With low digital literacy levels, weak digital concepts, and a lack of digitalization capabilities, the full benefits of digitalization in agriculture are difficult to achieve. The information barrier in agricultural product marketing remains intact, hindering progress [33]. Additionally, limited ability to diagnose, analyze, and collect digital information increases production decision-making obstacles, resource element mismatches, and natural and market risks in agricultural activities. This, in turn, increases agricultural production costs, reduces the willingness to adopt green technologies, and exacerbates agricultural carbon emissions. Based on this, Hypothesis 5 is proposed: the influence of digital rural construction on agricultural carbon emissions is threshold-based on rural human capital.

**Fig 1:**
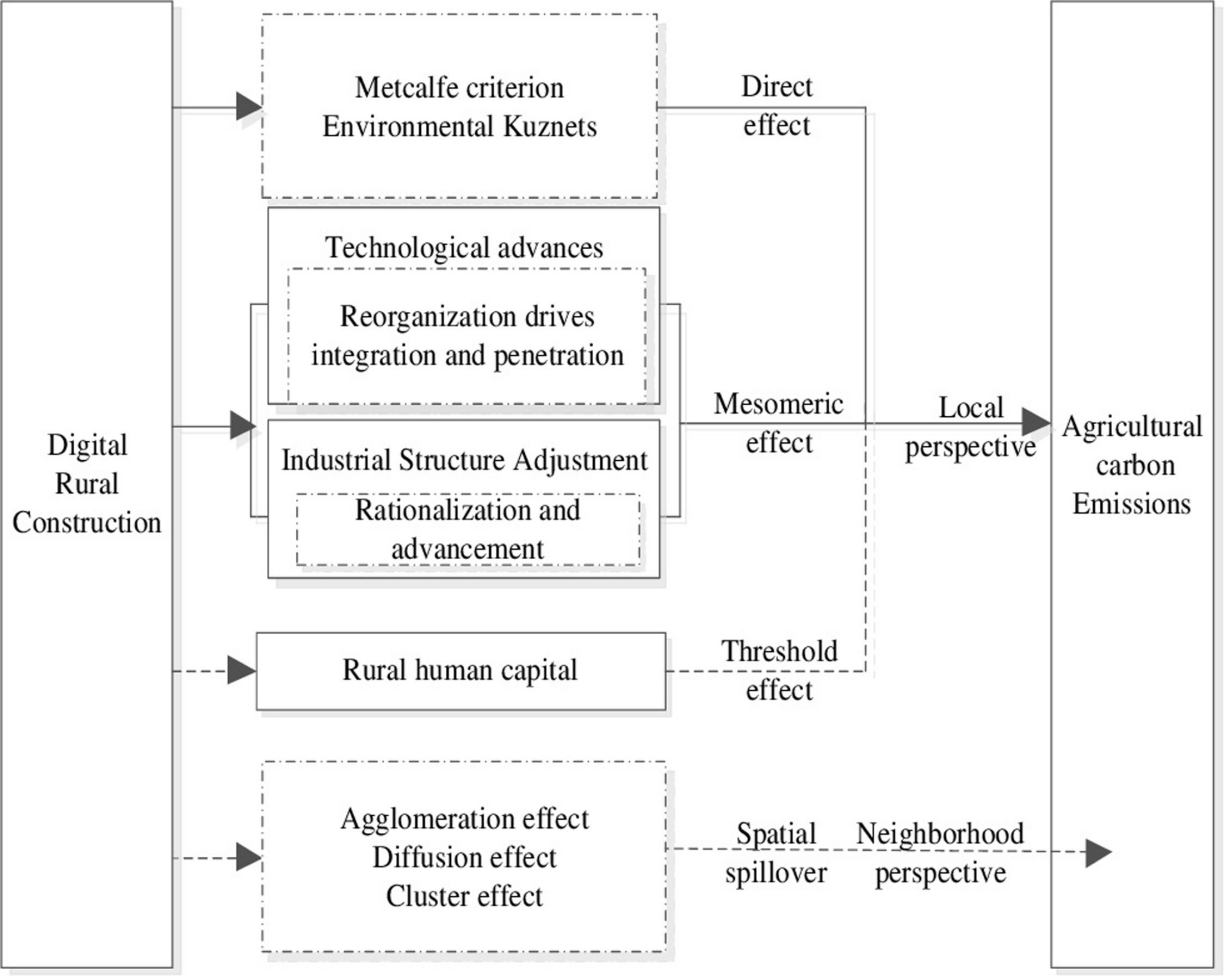
The theoretical analysis framework for the impact of digital rural construction on agricultural carbon emissions.

Based on comprehensive analysis, the theoretical framework for the impact of digital rural construction on agricultural carbon emissions was constructed, as shown in Fig 1.

## 3 Methods

### 3.1 Model construction

#### 3.1.1 Static panel model

In order to test the nonlinear impact mechanism of digital rural construction on agricultural carbon emissions, a static panel model is constructed as shown in Eq (1):

$$car_{it}=\alpha_{0}+\alpha_{1}dig_{it}+\alpha_{2}dig_{it}^{2}+\alpha_{3}con_{it}+\mu_{i}+\delta_{t}+\varepsilon_{it}$$ (1)

To further test the mediating mechanism of digital rural construction on agricultural carbon emissions, a three-step mediation model [34] is constructed based on (1), as shown in Eqs (2)–(4).


$$car_{it}=\alpha_{0}+\alpha_{1}dig_{it}+\alpha_{2}dig_{it}^{2}+\alpha_{3}con_{it}+\mu_{i}+\delta_{t}+\varepsilon_{it}$$ (2)

$$dm_{it}=\beta_{0}+\beta_{1}dig_{it}+\beta_{2}dig_{it}^{2}+\beta_{3}con_{it}+\mu_{i}+\delta_{t}+\varepsilon_{it}$$ (3)

$$car_{it}=\gamma_{0}+\gamma_{1}dig_{it}+\gamma_{2}dig_{it}^{2}+\gamma_{3}dm_{it}+\gamma_{4}con_{it}+\mu_{i}+\delta_{t}+\varepsilon_{it}$$ (4)

Where *car*_*it*_ is the agricultural carbon emission, *dig*_*it*_ is the level of digital rural construction, *con*_*it*_ is the control variable, *μ*_*i*_ represents individual fixed effects, *δ*_*t*_ represents time fixed effects, and *ε*_*it*_ represents random disturbance terms. *dm*_*it*_ are intermediate variables for the period t and represent agricultural green total factor productivity and agriculral structure adjustment, respectively.

#### 3.1.2 Spatial Durbin model (SDM)

To test spatial spillover effects, spatial autocorrelation test is conducted, and then a Spatial Durbin Model is constructed as shown in Eq (5):

$$\left\{\begin{array}{l} y_{it}=\beta x_{it}+\mu_{i}+\lambda_{t}+\varepsilon_{it}\\ \varepsilon_{it}=\theta \sum_{j=1}^{N}w_{ij}\varepsilon_{jt}+\xi_{it},\\ \xi ∼N(0,\sigma^{2}),\\ i=(1,2,\ldots N),t=(1,2,\ldots T) \end{array}\right.$$ (5)

In Eq (5), *y*_*it*_ represents agricultural carbon emissions. *w*_*ij*_ represents the elements in the spatial weight matrix W after undergoing normalization. ∑ *w*_*ij*_*y*_*it*_ represents the spatial lag variable of agricultural carbon emissions,it represents the impact of agricultural carbon emissions from neighboring provinces. *ρ* represents the spatial autoregressive coefficient, it represents the strength and direction of spatial autocorrelation under a given spatial structure, *x*_*it*_ represents digital rural construction, and *β* represents the impact coefficient, ∑ *w*_*ij*_*y*_*it*_ represents the spatial lag influencing variable of the explanatory variable of a province. *δ* represents the estimated spatial interaction influence coefficient. *μ*_*i*_ represents individual effects, *λ*_*t*_ represents time effects, ∑ *w*_*ij*_*ε*_*jt*_ represents spatial lag error variables, and *ξ*_*it*_ represents residual perturbation terms.*θ* represents the spatial error coefficient.

Anselin et al. [35]proposed that the estimation of parameters is difficult to reflect the marginal impact of the independent variable on the dependent variable due to the inclusion of both the dependent and independent spatial lag terms in the spatial Durbin model. On this basis, Lesage et al. [36] proposed a point estimation method based on the spatial Durbin model to measure spatial spillover effects, which is biased. Based on SDM, the partial differential method was used to divide spatial effects into direct and indirect effects, with the expression (6).


$$Y={\left(I-\rho w\right)}^{-1}\alpha +{\left(I-\rho w\right)}^{-1}(X^{'}\beta +wX^{'}\delta )+{\left(I-\rho w\right)}^{-1}\varepsilon$$ (6)

#### 3.1.3 Panel threshold model

To verify whether rural human capital disturbs the mechanism of the impact of digital rural construction on agricultural carbon emissions, according to Hansen [37], a panel threshold model was designed, using rural human capital as the threshold variable and the level of digital rural construction as the threshold dependent variable.

If the dataset is $\left\{y_{it},q_{it},x_{it},z_{it},1\leq i\leq N,1\leq t\leq T\right\},$, the single panel threshold model is Eq (7):

$$y_{it}=u_{i}+\beta_{1}x_{it}\times I(q_{it}\leq \gamma )+\beta_{2}x_{it}\times I\cdot (q_{it}\geq \gamma )+c_{it}z_{it}+\varepsilon_{it}$$ (7)

The triple panel threshold model is Eq (8):

$$y_{it}=u_{i}+\beta_{1}x_{it}\times I(q_{it}\leq \gamma_{1})+\beta_{2}x_{it}\times I(\gamma_{1}<q_{it}\leq \gamma_{2})+\beta_{3}x_{it}\times I(\gamma_{2}<q_{it}\leq \gamma_{3})+\beta_{3}x_{it}\times I(q_{it}>\gamma_{3})+c_{it}z_{it}+\varepsilon_{it}$$ (8)

Among them, *y*_*it*_ represents agricultural carbon emissions, *u*_*i*_ represents individual fixed effects, *x*_*it*_ represents threshold dependent variable, namely digital rural construction (dig), *q*_*it*_ refers to threshold variable, namely rural human capital (inc), and *z*_*it*_ refers to control variable. I *(·)* is an indicative function whose value is 1 or 0 based on the truth or falsehood in parentheses, *β*_*1*_*、β*_*2*_*、β*_*3*_ refers to the influence coefficient, γ is the threshold value, *ε*_*it*_ is an independent and identically distributed random perturbation term, where *i* represents the sample and *t* represents the time.

### 3.2 Variable definition

#### 3.2.1 Explanatory variable: Agricultural carbon emissions (car)

This study focuses on carbon emissions in the narrow sense of agriculture, also known as the planting industry, mainly derived from two aspects: firstly, the input of agricultural materials brings about carbon emissions, including fertilizers, pesticides, agricultural film, irrigation, diesel, etc. The second is the N_2_O emissions caused by the destruction of soil surface by planting, and the specific calculation formula is (9).


$$C=\sum C_{i}=\sum T_{i}\times \delta_{i}$$ (9)

In Eq (9), *C* represents the total amount of agricultural carbon emissions, *C*_*i*_ represents the carbon emissions brought by the type of carbon source, and *T*_*i*_ represents the number of *i* type of carbon sources and their carbon emission coefficients. The carbon source and emission coefficient of carbon emissions are determined based on relevant research conducted as Table 2. Meanwhile, Carbon emission intensity (car-s) is measured by the total agricultural carbon emissions/crop planting area.

**Table 2:** Carbon sources, emission coefficients, and sources of agricultural carbon emissions.

Carbon source	Indicator	Carbon emission coefficient	Source
Chemical fertilizer	Fertilizer application amount in the current year	0.8956kg·kg^-1^	Oak Ridge National Laboratory
Pesticide	Pesticide application amount in the current year	4.9341kg·kg^-1^	Oak Ridge National Laboratory
Agricultural film	Current application amount of agricultural film	5.1800kg·kg^-1^	Institute of Agricultural Resources and Ecological Environment, Nanjing Agricultural University
Diesel oil	Diesel fuel usage in the current year	0.5927kg·kg^-1^	IPCC
Agricultural irrigation	Irrigated Area	20.476kg/hm^2^	Adjust according to Dubey

#### 3.2.2 Core explanatory variable: Digital rural construction

Currently, there is no universally recognized evaluation system for the level of digital rural construction. Some scholars evaluate the digital rural construction from a single dimension, such as agricultural informatization [38], rural intelligence [39], and agricultural digitization [40]. Others construct indicator systems from multiple dimensions for comprehensive evaluation, such as evaluating the digital environment, input, service, and benefit [41], or evaluating the digital capital, industrial development, digital infrastructure, and digital service [42]. However, it is evident that current evaluation approaches tend to emphasize agriculture while neglecting the broader rural context, making it challenging to comprehensively and accurately assess the true level.

This study is based on the "Outline of digital rural construction Strategy" and follows the principles of scientificity, rationality, and obtainability of data. It takes into account both agricultural and rural digitization, including the digital infrastructure conditions. The study constructs a digital rural construction evaluation system consisting of three dimensions: digital basic environment, agricultural digitization, and rural digitization (refer to Table 3).The internal logic of the evaluation system is as follows: Firstly, The digital basic environment is considered a prerequisite for digital rural construction. It is primarily measured by factors such as 5G infrastructure, rural circulation facilities, agricultural electricity consumption, and the level of digital infrastructure construction. Secondly, Agricultural digitization is the core aspect of digital rural construction. It assesses the level of digitization in four key areas: agricultural production, sales, circulation, and service. This evaluation aims to comprehensively assess how digital technology is integrated into the agricultural industry chain. The degree of agricultural mechanization, scale of agricultural digitization, digitization of agricultural product transactions, and rural digital inclusive finance are used to gauge the level of digitization in these areas. Thirdly, Rural digitization represents the effectiveness of digital rural construction. It primarily focuses on the level of digitization in rural life. This is measured by indicators such as the prevalence of smartphones in rural areas, the penetration rate of the Internet, and the presence of rural meteorological observation stations. These indicators reflect the level of digital upgrade in communication, network, and weather services in rural areas. The application of digital technology in rural life is further measured by evaluating the per capita transportation and communication expenditures of rural residents.

**Table 3:** Evaluation index system of digital rural construction.

Primary indicators	Secondary indicators	Tertiary indicators	Description	Unit	Attribute
Digital rural constru- ction	Digital base environment	Construction of rural circulation facilities	Agricultural delivery route length	kilometer	+
		Number of 5G base stations	Total number of 5G base stations	individual	+
		Agricultural electricity consumption	Rural electricity consumption/ number of rural population	kwh/ person	+
		Digital base level	The proportion of Taobao villages in administrative villages	%	+
	Agriculture Digitization	The degree of mechanization of agricultural production	Total power of agricultural machinery/cultivated land area	kw/ha	+
		Digital scale in agriculture	Primary industry online retail sales/Number of rural population	million yuan/ person	+
		Agricultural digital service level	Rural Digital Inclusive Finance Index	%	+
		Digital trading of agricultural products	E-commerce retail sales	billion	+
	Countryside Digitization	Rural smartphone penetration	Rural mobile phone ownership per million households	division	+
		Rural transportation and communication expenses	Per capita transportation and communication expenses	yuan/person	+
		Rural Internet penetration	Number of rural Internet broadband access users	10,000 households	+
		Rural meteorological observation stations	Number of rural meteorological observation stations	piece	+

Furthermore, The entropy weight method is used to quantify the level of digital rural construction. Additionally, the study includes the square term of digital rural construction as represented by dig2 to examine the "inverted U-shaped" impact of digital rural construction on agricultural carbon emissions.

#### 3.2.3 Mediating variable

*(1) Technological progress*. The technological progress of agriculture is characterized by agricultural green total factor productivity (TFP), which is constrained by both resource elements and ecological environment in agricultural production systems. Following the research results of Guo and Liu [43], a measurement system of agricultural green total factor productivity that integrates "agricultural economy-resource-environment" is constructed, including:

Input indicators: select agricultural employment, calculated as the number of agricultural employees = first industry employment × (agricultural output value/agriculture, forestry, animal husbandry and fishing output value); crop sowing area; total agricultural machinery power; pure use of agricultural fertilizers; irrigated area and other indicators.

Expected output: select agricultural total output value, adjusted to constant prices in 2010.

Non-expected output: measure agricultural nonpoint source pollution, which is calculated according to the method of Guo and Liu [43]. In order to avoid the challenge of comparing agricultural green total factor productivity across different periods, use the global GML index to calculate agricultural green total factor productivity. Specific steps:

Firstly, construct the global benchmark production possibility set. Assuming that there are N inputs x in agricultural production, M expected outputs y, and O non-expected outputs b, the production possibility set p can be expressed as Eq (10):

$$P(x)=\left\{\begin{array}{l} (y^{t},b^{t}):\overset{I}{\underset{i=1}{\sum }}\lambda_{i}^{t}x_{i,n}^{t}\leq x_{n}^{t},n=1,2,\cdots ,N;\\ \overset{I}{\underset{i=1}{\sum }}\lambda_{i}^{t}y_{i,m}^{t}\geq y_{m}^{t},m=1,2,\cdots ,M;\\ \overset{I}{\underset{i=1}{\sum }}\lambda_{i}^{t}b_{i,O}^{t}=b_{O},o=1,2,\cdots O;\\ \lambda_{i}^{t}\geq 0;\;i=1,2,\cdots ,I; \end{array}\right\}$$ (10)

Secondly, calculate the direction distance function as shown in Eq (11).


$$D_{G}^{T}(x,y,t)=\max\left\{\beta |\left(y+\beta y,b-\beta b)\in P_{G}(x\right)\right\}$$ (11)

Where *β* represents the maximum expansion or contraction degree.

Third, calculate the GML index, as shown in Eq (12).


$$GML^{t,t+1}(x^{t},y^{t},b^{t},x^{t+1},y^{t+1},b^{t+1})=(1+D_{G}^{T}(x^{t},y^{t},b^{t})/(1+D_{G}^{T}(x^{t+1},y^{t+1},b^{t+1})$$ (12)

*(2) Agricultural structure adjustment*. Agricultural structure adjustment may result in either a reduction of emissions and an increase in carbon sinks, or an increase of emissions and a decrease in carbon sinks. The actual impact of agricultural structure adjustment needs to be verified. In this study, the proportion of total output value of planting industry to the total agricultural output value represents the agricultural structure adjustment, labeled as "str".

#### 3.2.4 Threshold variable

The rural human capital level (*jnx*) is used as the threshold variable. Since there is no direct data on rural human capital, it needs to be calculated using input-based, output-based, or education years-based methods. In this research, the education years-based method proposed by Hall and Jones [44] is used, as shown in Eq (13).


$$jnx_{i}=p_{0}\times 0+p_{1}\times 6+p_{2}\times 9+p_{3}\times 12+p_{4}\times 16$$ (13)

Among which, *jnx*_*i*_ indicates the average years of education. The current rural education level in China can be divided into five categories: below primary school, primary school, junior high school, senior high school (including secondary vocational education), and college and above, corresponding to the educational years of 0, 6, 9, 12, and 16 years, respectively. p0, p1, p2, p3, and p4 respectively represent the proportions of different educational levels among agricultural employment.

#### 3.2.5 Control variables

Agricultural carbon emissions are also influenced by macro-environmental and industrial factors. Therefore, the selected control variables are:

(1) Industrial concentration. The concentration of industries can measure the degree of specialization to a certain extent. Because location entropy can eliminate the impact of regional scale differences to some extent [45], regional entropy is selected to characterize (spe), expressed by Eq (14).

$$spe=\frac{g_{it}/a_{it}}{\sum_{i=1}^{n}g_{it}/a_{it}}\;$$ (14)

Where ’spe’ represents the regional entropy, *g*_*it*_ representing the grain output of the ith province or city in the tth year, and *a*_*it*_ representing the total agricultural output value of the ith province or city in the tth year.

(2) Fiscal support for agriculture (fin): Measured as a percentage of government financial expenditure allocated to agricultural, forestry, and water affairs.
(3) Urbanization level (urb): Measured as the proportion of permanent urban residents to the total population in a given area, expressed as a percentage.
(4) Agricultural disaster rate (diz): Measured as the proportion of disaster-affected farmland to the total area of cultivated land for crops, expressed as a percentage.
(5) Rural residents’ living standards (inc): Represented by the per capita disposable income of rural residents, and logarithmic transformation is used to address data balance issues.
(6) Crop rotation index (mul): The crop rotation index affects agricultural carbon emissions by influencing the scale of agricultural planting, and is represented as the ratio of the total area of cropped land to the total area of cultivated land.
(7) Agricultural land scale management level (sca): Measured by the grain crop planting area, and logarithmic transformation is used.

### 3.3 Data sources

A sample of 31 provincial regions was selected, excluding Hong Kong, Macau, and Taiwan, and data from 2011 to 2021 was collected from the China Statistical annual report, China Agricultural Statistical annual report, China Population and Employment Statistics annual report, and various provincial statistical annual reports, as well as the National Bureau of Statistics website and Guotai An database. Linear interpolation was used to fill in missing data. To avoid multicollinearity, variance inflation factor (VIF) tests were conducted, resulting in VIF values below 10 and tolerances above 0.1, indicating the absence of multicollinearity.

The descriptive statistics of all variables are shown in Table 4. The average level of the digital rural construction index is 0.620, indicating a generally low level across the sample. Agriculture carbon emissions range from a minimum of 14.310 million tons to a maximum of 995.600 million tons, exhibiting significant regional differences. The average value of agricultural green total factor productivity is 1.035, indicating an overall improvement. The standard deviation of agricultural structure adjustment index is 8.461, reflecting significant regional differences. Rural human capital ranges from a minimum of 3.820 to a maximum of 9.800, highlighting significant disparities across different regions. The polarization of control variables is also evident.

**Table 4:** Descriptive statistical results.

Variable	Number	Mean	Standard error	Minimum	Maximum
*dig*	341	0.620	0.155	0.250	0.966
*car*	341	328.100	233.200	14.310	995.600
*tfp*	310	1.035	0.070	0.749	1.498
*ind*	341	52.300	8.461	30.200	72.110
*jnx*	341	7.680	0.820	3.820	9.800
*spe*	341	3.226	1.997	0.513	13.180
*fin*	341	11.580	3.394	4.110	20.380
*urb*	341	58.640	13.070	22.81	89.600
*diz*	341	14.270	11.600	0.000	69.590
*inc*	341	9.390	0.433	8.271	10.560
*sca*	341	7.651	1.330	3.840	9.585
*mul*	341	66.080	14.510	35.510	97.080

**Fig 2:**
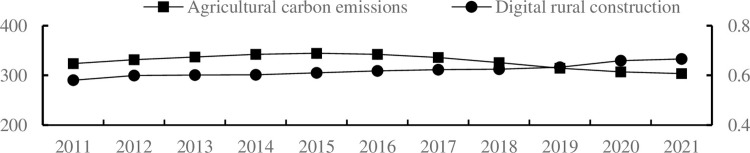
The trend of variables. The level of digital rural construction can be found on the secondary coordinate axis.

From the trend of changes in digital rural construction and agricultural carbon emissions (see Fig 2), it can be seen that digital rural construction shows a significant growth trend, while agricultural carbon emissions show a trend of first increasing and then decreasing. This to some extent indicates that the impact of digital rural construction on agricultural carbon emissions is non-linear, and the specific impact mechanism needs further testing.

## 4 Results

### 4.1 Regression results

Based on Eq (1), parameter estimation was conducted. LM test, F test, and Hausman test were performed, and the fixed-effect model was chosen to analyze the direct effect of digital rural construction on agricultural carbon emissions. The results are presented in Table 5.

**Table 5:** The results of benchmark model.

Variable	(1)	(2)	(3)	(4)
	car	car	car	car
*dig*	0.336^***^	0.259^*^	0.443^***^	0.305^***^
	(0.054)	(0.143)	(0.074)	(0.031)
*dig2*	-	-	-0.409^*^	-0.394^*^
	-	-	(0.215)	(0.231)
*Controls*	No	Yes	No	Yes
*Year*	Yes	Yes	Yes	Yes
*Province*	Yes	Yes	Yes	Yes
*N*	341	341	341	341
*R* ^*2*^	0.356	0.332	0.368	0.457

Note:

^***^ p<0.01

^**^ p<0.05

^*^ p<0.1,Standard error in parentheses.

Column (1) displays the estimation results without the quadratic term of digital rural construction and without control variables. Column (2) shows the results without the quadratic term of digital rural construction but with control variables. Column (3) presents the results with the quadratic term of digital rural construction but without control variables. Column (4) presents the results with both the quadratic term of digital rural construction and control variables added. It was found that the regression coefficient of digital rural construction is significantly positive in all four cases but becomes significantly negative after the quadratic term is added. Furthermore, the coefficients of digital rural construction and its quadratic term are somewhat reduced after adding control variables, indicating some level of robustness in the model. Among the four cases, Column (4) demonstrates the best fit of the model. The coefficient of the first-order term of digital rural construction is significant and positive, while the coefficient of the quadratic term is significant and negative. This suggests an "inverted U-shaped" relationship between digital rural construction and agricultural carbon emissions, thus supporting Hypothesis 1. There seems to be no linear correlation between digital rural construction and agricultural carbon emissions. This can be attributed to the ongoing rapid advancement of digital rural construction, comprehensive deployment of digital infrastructure, and the increased energy consumption of electricity and coal in the process, leading to a rise in total agricultural carbon emissions and reducing the carbon reduction effect of digital rural construction. However, leveraging digital agricultural technology, digital rural construction is integrating into the production, operations, and management system of agricultural products, driving the diffusion of green technology and knowledge, thereby reducing carbon emissions.

### 4.2 Impact mechanism analysis

According to theoretical analysis, digital rural construction can have an impact on agricultural carbon emissions by influencing technological progress and agricultural structure adjustment. In order to examine the mediating effect, a test is conducted using Eqs (2)–(4), and the results are presented in Table 6.

**Table 6:** Mediation effect test.

Variable	(1)	(2)	(3)	(4)	(5)	(6)
	*car*	*tfp*	*car*	*ind*	*car*	*car*
*dig*	0.305^***^	0.361^*^	0.284^***^	0.438^*^	0.261^*^	0.219^*^
	(0.031)	(0.202)	(0.026)	(0.231)	(0.141)	(0.115)
*dig2*	-0.394^*^	0.078	-0.339^*^	0.236	-0.372^*^	-0.206^*^
	(0.231)	(0.072)	(0.185)	(0.582)	(0.195)	(0.107)
*tfp*	-	-	-0.269^**^	-	-	-0.167^**^
	-	-	(0.116)	-	-	(0.073)
*ind*	-	-	-	-	-0.301^*^	-0.148^**^
	-	-	-	-	(0.163)	(0.059)
*Controls*	Yes	Yes	Yes	Yes	Yes	Yes
*N*	341	341	341	341	341	341
*R* ^*2*^	0.457	0.355	0.332	0.304	0.358	0.462

Note:

^***^ p<0.01

^**^ p<0.05

^*^ p<0.1,Standard error in parentheses.

#### 4.2.1 The mediating effect of technological progress test

According to the findings presented in Table 6, the relationship between digital rural construction and agricultural carbon emissions follows an inverted-U shape in column (1). However, in column (2), digital rural construction is found to positively contribute to the improvement of agricultural total factor productivity without displaying an inverted-U shape. Furthermore, comparing the coefficients in column (3) to those in column (1), it is observed that the coefficients in column (3) have decreased. This, along with the results in column (6), suggests that agricultural total factor productivity partially mediates the nonlinear relationship between digital rural construction and agricultural carbon emissions, thereby validating hypothesis 2.

Tracing the root cause, The integration of internet technology, digital technology, and information technology with traditional agricultural and rural production systems comprises digital rural construction. As digital rural construction is implemented nationwide, the collection, transmission, and processing of digital information have significantly improved. The decreasing cost of digital technology, in accordance with the law of diminishing returns, has promoted the dissemination and advancement of green technology. This, in turn, has increased the enthusiasm of agricultural operators towards implementing new green and low-carbon technologies and stimulated the development of informal environmental regulations. Consequently, agricultural green technology has progressed, leading to optimized resource allocation, economies of scale, upgraded operational modes, improved agricultural total factor productivity, and reduced agricultural carbon emissions.

#### 4.2.2 The mediating effect of agricultural structure adjustment test

According to column (4) in Table 6, digital rural construction promotes agricultural structure adjustment. Comparing column (1) and column (5), with the addition of agricultural structure adjustment variable, the linear and quadratic coefficients of digital rural construction both decrease to some extent, but their significance does not change. Further combined with column (6), with the addition of the variables of agricultural green total factor productivity and agricultural structure adjustment, the significance of the linear and quadratic fitting coefficients of digital rural construction does not change fundamentally. Therefore, agricultural structure adjustment also plays a partial mediating role in the nonlinear impact of digital rural construction on agricultural carbon emissions, and hypothesis 3 is validated. This suggests that digital rural construction leverages the penetration effect of the digital economy to generate positive externalities, and exerts a positive pull effect on agricultural structure adjustment, driving the agricultural structure towards digitalization, greenization, and high-end development, reducing the dependence on high-energy consumption and pollution-intensive factors, thereby reducing agricultural carbon emissions. Additionally, digital rural construction also promotes the superimposition of digital factors with traditional production factors such as labor, capital, and land, promoting the rationalization and low-carbon adjustment of agricultural structure, reducing resource consumption, improving efficiency, and reducing costs, thus promoting carbon emission reduction. Based on these findings, it should fully leverage the structural effects of digital rural construction, increase the penetration of the digital rural platform in the agricultural field, and promote the full deployment of "digital+" agricultural new formats such as digital planting, online monitoring, and intelligent water and fertilizer integration, thereby reducing agricultural carbon emissions.

### 4.3 Spatial spillover effects test

The spatial correlation of core variables was tested first (see Table 7) to test the spatial spillover effects. The results show that both digital rural construction and agricultural carbon emissions have positive spatial correlation, indicating a need to further explore the spatial effects.

**Table 7:** Moran index of digital rural construction and agricultural carbon emissions.

Year	Moran’I-car	p	Moran’I-dig	p
2011	0.234	0.007	0.354	0.002
2012	0.223	0.010	0.315	0.011
2013	0.212	0.013	0.301	0.003
2014	0.188	0.023	0.306	0.004
2015	0.177	0.028	0.397	0.021
2016	0.170	0.032	0.399	0.023
2017	0.169	0.034	0.401	0.016
2018	0.167	0.035	0.406	0.015
2019	0.159	0.040	0.415	0.014
2020	0.163	0.037	0.423	0.007
2021	0.164	0.036	0.433	0.006

After conducting LM and HAUSMAN tests, as well as comparisons using Schwarz criterion (SC), Akaike information criterion (AIC), and log-likelihood ratio ln(L), a spatial Durbin two-way fixed-effects model was chosen to examine the spatial spillover effects of digital rural construction on agricultural carbon emissions. To ensure the accuracy of the results, spatial weights matrices were used with adjacency and geographical distance to test the spatial effects(see Table 8).

**Table 8:** The results of spatial effect test.

Variable	Adjacency matrix w1			Geographic distance weight matrix w2		
	Main	Direct	Indirect	Main	Direct	Indirect
*dig*	0.636^***^	0.638^***^	0.201^***^	0.661^***^	0.667^***^	0.262^***^
	(0.103)	(0.026)	(0.011)	(0.052)	(0.055)	(0.021)
*dig* ^*2*^	-0.525^***^	-0.517^***^	-0.132^***^	-0.516^***^	-0.508^***^	-0.152^***^
	(0.043)	(0.027)	(0.004)	(0.123)	(0.017)	(0.013)
*Controls*	yes			yes		
*ρ*	0.289^***^			0.291^***^		
	(0.062)			(0.081)		
*N*	341			341		
*R* ^*2*^	0.418			0.401		

Note:

^***^ p<0.01

^**^ p<0.05

^*^ p<0.1,Standard error in parentheses.

Based on the coefficients and significance levels of both direct and indirect effects, the study found that digital rural construction had an inverted U-shaped relationship with respect to local agricultural carbon emissions, and showed similar effects on adjacent and geographically close areas. The effects of digital rural construction on adjacent and geographically close areas were also similar, suggesting the presence of spatial spillover or a "neighborhood effect." Therefore, Hypothesis 4 was confirmed. Additionally, the coefficients of the direct effects were higher than those of the indirect effects. This suggests a negative correlation between the carbon emission reduction of digital rural construction and spatial distance. These findings highlight the potential of digital rural construction to optimize resource allocation, reduce agricultural carbon emissions, and promote green and coordinated regional agriculture construction. It is important to collaborate with neighboring regions to enhance the spatial spillover effects of carbon reduction and create a ripple effect. While promoting digital rural construction locally, attention should also be given to neighboring regions to achieve sustainable regional development.

### 4.4 Threshold effect test

The role of digital rural construction in promoting agricultural carbon emissions reduction is not just limited to material support, such as digital infrastructure and mechanical equipment, but also depends on intellectual support, such as human knowledge and digital literacy. In order to analyze the threshold effect of rural human capital in the impact of digital rural construction on agricultural carbon emissions, it is assumed the existence of three threshold levels and conducted 300 bootstrap iterations for significance testing using Eqs (7) and (8). However, only a single threshold level passed the significance test, as shown in Table 9. It then used the LR test method to determine the threshold value of rural human capital, which was found to be 8.830, as presented in Table 10. Further regression analysis was then conducted using a single threshold model. The study sample was divided into two intervals: below and above the threshold value. The results of this regression analysis are shown in Table 11.

**Table 9:** Threshold effect test results.

Model	F	p	Crit10	Crit5	Crit1
Single threshold	41.380	0.063	29.157	47.113	103.674
Double threshold	3.980	0.680	18.219	29.161	101.938
Triple threshold	2.730	0.883	22.446	37.518	51.338

**Table 10:** Threshold estimation results.

Threshold type	Threshold	95% lower confidence limit	95% Confidence Upper Limit
First threshold	8.830	8.780	8.880

**Table 11:** Single threshold regression.

Variable	(1)
	car
dig(jnx≦8.830)	0.151^***^
	(0.013)
dig(jnx>8.830)	-0.524^***^
	(0.112)
Observations	341
R^2^	0.458

Note:

^***^ p<0.01

^**^ p<0.05

^*^ p<0.1,Standard error in parentheses.

Research findings indicate that when the rural human capital level is below 8.830, the impact coefficient of digital rural construction on agricultural carbon emissions is positive and statistically significant at the 1% level. It suggests that in areas with lower human capital levels, digital rural construction exacerbates agricultural carbon emissions. This can be attributed to inadequate knowledge and understanding of digital technologies and green practices among agricultural operators. The limited acceptance and adoption of agricultural green technology negatively impact decision-making efficiency and hinder the development of an industry structure focused on advancement and green low-carbonization. Consequently, total agricultural carbon emissions increase.

Conversely, when rural human capital exceeds the threshold level of 8.830, the impact coefficient of digital rural construction on agricultural carbon emissions becomes significantly negative. As rural human capital improves, agricultural operators gain a better understanding of the benefits of digital rural construction. This leads to increased adoption of digital agriculture technology and the application of green low-carbonization knowledge. The prevalence and high-level popularization of agricultural digitalization is promoted, resulting in improved efficiency in agricultural green production and a reduction in agricultural carbon emissions.

To fully realize the potential of digital rural construction in reducing agricultural carbon emissions, it becomes essential to implement various measures aimed at improving the level of rural human capital. Therefore, it is crucial to verify hypothesis 5, which suggests that enhancing rural human capital is necessary for maximizing the agricultural carbon emissions reduction effect of digital rural construction.

### 4.5 Robustness test

#### 4.5.1 Endogeneity discussion

To address potential endogeneity problems, the fixed effects model was employed to account for unobservable variables that remain constant over time. Additionally, control variables were included to mitigate endogeneity issues arising from missing variables. However, the benchmark regression results indicated a reciprocal relationship between digital rural construction and agricultural carbon emissions. This bidirectional causal relationship introduces estimation bias due to endogeneity. To mitigate these concerns, the lag one stage processing method and instrumental variable approach were adopted (see Table 12).

**Table 12:** Endogeneity and robustness test.

Variable	(1)	(2)	(3)	(4)	(5)	(6)
	System GMM	IV-2SLS		Replace the dependent variable	Excluding samples	Shorten sample period
	*car*	L-1	L-2	*car-s*	*car*	*car*
*dig*	-	-	0.315^***^	0.329^***^	0.381^***^	0.335^***^
	-	-	(0.012)	(0.025)	(0.011)	(0.016)
*dig2*	*-*		-0.368^***^	-0.392^***^	-0.377^***^	-0.363^***^
			(0.015)	(0.021)	(0.034)	(0.022)
*L1*.*dig*	0.327^***^	-	-	-	-	-
	(0.014)	-	-	-	-	-
*L1*.*dig2*	-0.384^***^	-	-	-	-	-
	(0.023)	-	-	-	-	-
*L3*.*dig*	-	0.296^***^	-	-	-	-
	-	(0.037)	-	-	-	-
*L3*.*dig2*	-	-0.277^***^	-	-	-	-
	-	(0.013)	-	-	-	-
*urp*	-	0.326^***^	-	-	-	-
	-	(0.024)	-	-	-	-
*Controls*	Yes	Yes	Yes	Yes	Yes	Yes
*Year*	Yes	Yes	Yes	Yes	Yes	Yes
*Province*	Yes	Yes	Yes	Yes	Yes	Yes
AR(2)	0.195	-	-	-	-	-
Sargan test	31.276	-	-	-	-	-
	[1.000]	-	-	-	-	-
Kleibergen Paap rk LM	-	-	67.346	-	-	-
	-	-	[0.000]	-	-	-
Cragg-Donald Wald F	-	-	439.293	-	-	-
	-	-	{16.380}	-	-	-
Hansen-p	-	-	0.975	-	-	-
*N*	311	280	280	341	297	279
*R* ^2^	0.458	0.422	0.439	0.428	0.442	0.369

Note: The value in square brackets is the p-value, and the value in the curly bracket is the critical value corresponding to the 5% level of the Stock Yogo test.

(1) One phase lag in digital rural construction. Considering the possibility of time lag in digital rural construction, lag-one digit (L1.dig) is handled for digital rural construction, and the system GMM method is used for re-estimation. The results are shown in column (1) of Table 12, where AR(2) and Sargan test results show that there is no second-order serial correlation and over-identification problem, and the GMM regression results are reliable. It can be seen that the influence of lag-one digital rural construction on agricultural carbon emissions still shows a "inverted U-shaped" pattern, and is significant at the 1% level of significance test, indicating that lag-one digital rural construction still has a carbon emission reduction effect.

Instrumental variables method. On the one hand, digital rural construction cannot be achieved without internet technology, which originated from fixed telephone lines. Therefore, areas with high fixed telephone penetration rates in history also tend to have higher internet penetration rates, which is an important prerequisite for digital rural construction. Based on this, the number of fixed telephones per ten thousand people in 1998 is selected as the instrumental variable, which satisfies the requirement of correlation and meets the exogeneity requirement due to the rapid development of the internet technology and the continuous penetration of digital technology. However, as the number of fixed telephones per ten thousand people in 1998 is a cross-sectional data, it cannot perform panel data fixed effect analysis. Therefore, following the approach of Nunn and Qian [46], the interaction term (urp) between the number of fixed telephones per ten thousand people in 1998 and the previous year’s urban internet broadband penetration rate is used as a measurement indicator for the instrumental variable. On the other hand, the lagged three periods of digital rural construction (L3.dig) are selected as the instrumental variable. Digital rural construction is a systematic project that requires continuous promotion and is conducive to laying a good foundation for later development and meets the requirement of correlation. The relationship between the lagged three periods of digital rural construction and the current digital rural construction is insignificant, thus having the exogeneity feature. The IV-2SLS method was used to reexamine the relationship between digital rural construction and agricultural carbon emissions. The results, presented in columns (2)-(3) of Table 12, provide insights into this mechanism. The Kleibergen-Paap rk LM test value is 67.346, which surpasses the 1% significance level, indicating that there are no unidentifiable problems in the model. The Cragg-Donald Wald F value is 439.293, exceeding the critical value of 16.380 at the 5% significance level. This suggests that there is no issue of weak instrument problem, reinforcing the reliability of the instrument variables. Additionally, the Hansen J statistic’s p-value is 0.975, indicating that the exogeneity requirement is satisfied by the instrument variables. Considering the results in column (3), it can be inferred that the impact of digital rural construction on agricultural carbon emissions, after addressing endogeneity concerns through the instrumental variable approach, remains similar to the baseline regression results. This suggests the robustness of the findings.

#### 4.5.2 Robustness re-examination

To ensure the reliability of the findings, two approaches were employed for further examination. Firstly, the dependent variable was replaced with agricultural carbon emission intensity (car-s). The results, presented in column (4) of Table 12, indicate that the outcomes remained largely unchanged. Secondly, samples from the four municipalities under the direct jurisdiction of the central government (Beijing, Shanghai, Tianjin, and Chongqing) were excluded from the samples, considering the unclear distinction between urban and rural areas and their advanced digital infrastructure compared to other provinces, the column (5) of Table 12 displays the re-evaluated results, which exhibited no significant alterations. Furthermore, the sample period was shortened to address the influence of the COVID-19 pandemic, covering the years 2011–2019. The re-regression results, presented in column (6) of Table 12, showed no substantial divergence from the baseline regression findings. As a result, it can be concluded that the agricultural carbon reduction effect of digital rural construction remains robust.

## 5 Discussion

### 5.1 Built of an integrated evaluation system for digital rural construction

The current state of the comprehensive indicator system for measuring the level of digital rural construction is lacking in several aspects. Firstly, it primarily focuses on providing theoretical explanations without sufficient measurable indicators. Additionally, there is an inadequate coverage of digital rural indicators. Many analyses conducted in this field tend to approach it from a specific perspective, resulting in a limited understanding. For instance, Zhu et al. [42] select evaluation indicators from the input-output perspective, while Zhang et al. [40] choose indicators from the perspective of digital agriculture. In contrast, this study aims to address these limitations by developing an evaluation system that encompasses three dimensions: digital infrastructure, agricultural digitalization, and rural digitalization. By considering these dimensions, this study takes into account the essential attributes of digital rural areas. The evaluation system is designed to encompass prerequisite conditions, core content, and outcomes of digital rural construction. The selection of indicators for this evaluation system is reliable and effective in measuring the progress and effectiveness of digital rural construction.

### 5.2 The multi-dimensional explanation of the impact mechanism and pathways of digital rural construction on agricultural carbon emissions

Existing research has primarily focused on the positive role of digital rural construction in promoting agricultural development and exploring the impact mechanisms of agricultural carbon emissions. However, there is a lack of research examining the mechanisms of carbon emission reduction in agriculture from the perspective of digital rural construction. This study aims to fill this gap by investigating the non-linear and spatial spillover effects of digital rural construction on agricultural carbon emission reduction.

The study also examines the impact pathways of rural human capital on the threshold and tests the differentiated impact mechanisms of different levels of digital rural construction on agricultural carbon emission reduction under dual carbon goals. The study reveals three important findings. Firstly, digital rural construction has a non-linear impact on agricultural carbon emissions by promoting green total factor productivity and agricultural structure adjustment. This finding aligns with previous research by Du et al. [16], but adds that green total factor productivity acts as a mediator and has a positive role in the impact of digital rural construction on agricultural carbon emissions.

Secondly, there is a threshold effect in the impact of digital rural construction on agricultural carbon emissions, with rural human capital being the sole threshold. This finding contrasts with the research by Fu et al. [47], emphasizing the significant moderating role of rural human capital in the relationship between digital rural construction and green agricultural development. This highlights the importance of rural human capital in the mechanism of digital rural construction’s impact on agricultural carbon emissions.

Thirdly, digital rural construction demonstrates an "inverted U-shaped" effect on agricultural carbon emissions locally, with initial promotion followed by suppression. It also exhibits spatial spillover effects on agricultural carbon emissions in adjacent areas, with initial promotion followed by suppression. These findings differ from previous studies conducted by Liu et al. [1], Du et al. [16], and Fu et al. [47]. The study further investigates the impact mechanism of digital rural construction on agricultural carbon emissions from a spatial perspective, providing valuable references for promoting coordinated digital rural construction across regions.

Overall, this study contributes to understanding the impact of digital rural construction on agricultural carbon emissions and provides insights for promoting sustainable agricultural practices through digital rural construction.

### 5.3 Limitations and future research

This study not only provides new ideas for exploring carbon reduction paths and fully releasing the dividends of digital rural construction, but also expands the dynamic contextual research on the impact of digital rural construction on carbon emissions and deepens the theory of agricultural green development. However, there are still certain limitations. Firstly, the evaluation of digital rural construction is a complex system engineering and involves numerous complex factors, which poses a great challenge to the evaluation of the effectiveness and level of digital rural construction. This study only selected indicators from the three dimensions of "infrastructure-agriculture-rural" to construct the evaluation index system. Secondly, due to the availability of data, the time limit of this study is only 11 years, and panel data at the provincial level was used. However, the digital rural construction action plan was promoted at the national level in 2018, and the carbon reduction mechanism of digital rural construction still has certain dynamic properties which have not been deeply discussed. Thirdly, this study focuses on the scale and intensity of the impact of digital rural construction on agricultural carbon emissions, but its impact on efficiency has not been explored. Therefore, future research can delve deeper into the subject matter from the perspective of systemic theory, temporal and spatial dynamic evolution, and carbon emissions efficiency.

## 6 Conclusion

It is important to fully tap into the carbon reduction effect of digital rural construction in agriculture under the dual carbon goal to break the constraints of agricultural economy and resource environment and promote rural revitalization for achieving agricultural and rural modernization. In order to investigate whether digital rural construction in agriculture leads to "dividend" or "disadvantage" in carbon reduction, a panel dataset of provincial level covering the period from 2011 to 2021 is utilized. A comprehensive evaluation index system is constructed based on dimensions including digital infrastructure, agricultural digitization, and rural digitization. Through empirical analysis from multiple perspectives such as nonlinear effect, mediation effect, spatial spillover effect, threshold effect, and heterogeneity, the impact mechanism of digital rural construction on agricultural carbon emissions is examined. The conclusion is as follows: First, the impact of digital rural construction on agricultural carbon emissions shows a non-linear "inverted U-shaped" feature, and this conclusion still holds after a series of robustness tests such as considering endogeneity, replacing dependent variables, excluding municipalities, and shortening the sample period. Second, digital rural construction has a non-linear impact on agricultural carbon emissions by promoting green total factor productivity and agricultural structure adjustment. Third, Digital rural construction has a dual effect on local agricultural carbon emissions in terms of both promoting and then restraining the emissions, which has a spatial spill-over effect in the neighboring areas. Fourth, there is a threshold effect of rural human capital in the impact process of digital rural construction on agricultural carbon emissions. After crossing the threshold value of 8.830, the carbon reduction effect becomes prominent. Overall, these findings highlight the importance of maximizing the carbon reduction potential of digital rural construction in achieving the dual carbon goals.

Based on the above conclusions, there are several policy recommendations.(1)Continuously promote digital rural construction and consolidate the infrastructure of digital rural construction. Based on new infrastructure, establish and expand 5G network coverage in rural areas, vigorously promote the digital transformation of rural infrastructure, fully promote the transformation and upgrading of rural power grids, and deepen the "information entering villages and households" project.(2)Steadily promote the improvement of agricultural green total factor productivity and promote the advanced development of the agricultural industry structure. It is necessary to further break through the green core technology of key areas in agriculture, vigorously support agricultural innovation, promote the upgrading of green technology progress, and promote the deep integration of digital technology and traditional agriculture. It is also necessary to continuously improve the environmental regulatory system of agriculture, construct a systematic agricultural ecological compensation mechanism, and promote the development of the agricultural industrial chain towards green and low-carbon.(3) Play a significant role in spillover effects and promote regional sharing of digital rural construction dividends. At the national level, a digital rural construction collaborative promotion system should be established for regional cooperation coordinated by the central government, encouraging regions with relatively backward levels of digital rural construction to take full advantage of the series of favorable policies such as new infrastructure and east-west calculation, and promoting the "enclave" form of regional digital rural construction. At the regional level, strengthen experience exchange and sharing of digital rural construction between regions. (4)Fully cultivate rural digital human capital and empower the development of agricultural green, low-carbon transition. On the one hand, establish a digital technology training system for agriculture. Focus on promoting the participation of new agricultural personnel, family farms, and cooperatives in training sessions to promote a "ripple effect." On the other hand, carry out a special action to attract digital benefits to agriculture.

## Supporting information

S1 File (DOCX)

S1 Data (XLSX)
