# Supplementary material for: The agricultural carbon reduction effect of digital rural construction under the dual carbon target
Source: PLoS One. 2024 Apr 10;19(4):e0299233. doi: 10.1371/journal.pone.0299233 (PMC11006124; doi:10.1371/journal.pone.0299233)
Supplement: S1 File — (DOCX) [file pone.0299233.s001.docx]

DEAR:

Please help me to revise the Funding Information to "Funder Name: National Social Science Foundation"Research on the Convergence and Impact Mechanism of Agricultural Green Total Factor Productivity in the Context of Food Security" Grant Number: 21BGL160, Grant Recipient: professor haihong guo.; Funder Name: Shandong Province Soft Science Key R&D Plan (Soft Science) Key Project, "Research on the Development Path of Green and Low Carbon Transformation in Agriculture in Shandong Province”,Grant Number: 2023RZB06045, Grant Recipient: professor haihong guo.

Please help me to revise Financial Disclosure statement to"This work was supported by [National Social Science Foundation"Research on the Convergence and Impact Mechanism of Agricultural Green Total Factor Productivity in the Context of Food Security"(Grant numbers:21BGL160),and Shandong Province Soft Science Key R&D Plan (Soft Science) Key Project "Research on the Development Path of Green and Low Carbon Transformation in Agriculture in Shandong Province (Grant numbers :2023RZB06045)]. The funders had no role in study design, data collection and analysis, decision to publish, or preparation of the manuscript."

THANK YOU VERY MUCH !

Haihong GUO
